# A Provident Out-Patient Department

**Published:** 1890-05-17

**Authors:** 


### A Provident Out-Patient Department.

By the Right Hon. the Earl of Derby, K.G.

Speaking at the Festival Dinner of the Metropolitan Hospital, the Earl of Derby, K.G., said that it had become a commonplace to say—he had said it himself many times, and he had heard it many times more—that, of all forms of charity, hospitals were at once the most certain to be useful, and the least liable to be abused. There was practically next to no danger of their being imposed upon when medical relief was in question. The illness of a patient was a fact, whatever his merits or demerits might be, however worthless he might be himself, however little valuable his life might be to anybody else, still, he was a patient, and must have his arm or leg set if they were broken, or his complaint attended to if he was ill. Humanity required that, and no one would contend that because his antecedents were unsatisfactory he ought to be left to suffer unrelieved. Then there was the fact with which they were all familiar, that hospitals were schools of medical and surgical science, by which no class profited more than the class which never entered those hospitals, for the lessons learned there were utilised in private practice. He would not labour that point, because they were all agreed that by some means, private or public, the great London hospitals had to be kept up. The only question was—How? If voluntary aid were to fail they would have to fall back on the rates and taxes, and, with all due respect to our present rulers, the rates and taxes were now high enough in all conscience without adding to them. But voluntary aid would not fail unless the English character altered more than it was likely to do. Whatever might be the faults of the present age, it was certainly not one in which indifference or apathy for the suffering of the poorer classes were common defects. He should say that never in our history, perhaps never in the history of the world, had there been so earnest and genuine a wish to improve the condition of those who suffered; and if we failed in any respect it was not from want of sympathy, but rather from a misplacement of sympathy; it was rather because we were too easily led away to adopt any scheme that had about it an air of benevolence, without always inquiring whether it would do good instead of harm. He had no fear, therefore, that our great hospitals would not continue to be supported, and he repeated that of all charities they were the least liable to be abused.

But there certainly was one abuse to which they were liable, and one which had long been injurious, and against that it was the great object of all hospital authorities to provide what might, at least, be a partial remedy. The common practice of hospitals was to open their doors freely to patients who came provided with letters of introduction; in some to those who came without such introduction; and always, of course, to accidents. Now, the disadvantage of the letter system was that it virtually closed the hospital to those who had not some friends amongst subscribers and donors—that was to say, it closed the doors against those who, in all probability, needed assistance the most. A countervailing advantage to that was the fact that it gave to the subscribers a certain return for their contributions, and so encouraged others to contribute. Under an absolutely free system of admission, the difficulty was of a different kind. It was this, that all could not be helped, and there was no easy or simple rule by which deserving and undeserving cases could be discriminated. Under both these systems there was the same difficulty that the help given, whether it be given as a matter of personal favour or on the general ground of humanity, is entirely gratuitous, and it was notorious that many persons had no shame in receiving gratis medical relief which they were well able to pay for, who yet would not stoop to ordinary begging. It was not good to create in the mind of the poorest man the idea that whenever he might be ill he had a right to be cured at other people's expense. How was the difficulty to be met? The Committee of the Metropolitan Hospital thought it was best met by going on the good old rule of helping those who helped themselves. They quite admitted that a great number of the working classes could not be expected to pay the whole cost of a long illness; but, because something was done for them, that was no reason why they should not be asked, on their side, to do what they reasonably could be expected to do. The Committee believed that the better sort of working men would accept that proposition as fair and just, especially as its object was not to save the pockets of rich people, but to make the money of the contributors go further than it otherwise would. The plan adopted, as he understood it, was to ask those who were willing to join the Provident Department to pay a very small weekly or monthly contribution, not enough to be felt as a burden, but giving the contributor a right to relief from the hospital, and enabling him to feel that he was not merely the recipient of charity. He thought there was no question but that the principle thus laid down was a good one if only it would work. It was exposed here in London to a severe test, for they were asking people to pay in part for that which they had been accustomed to get for nothing—in fact, for that which they did get for nothing in other localities of the town. It would be impossible, he thought, to try an experiment under circumstances more arduous and more discouraging. Yet that experiment, so far as it had gone, had been entirely successful. It was stated at last year's dinner that 11,000 persons had placed themselves under the new arrangement. He was informed that at the present time the number had increased to 16,000. The fact was that men of the better sort in any class, though they might readily accept help, and they were quite right to do so, which they could not do without, had a strong sense of independency. They disliked anything that looked like pauperism, and welcomed the opportunity given them of receiving hospital relief as a right which they had partially paid for and not as a favour for which they had to beg. He would not, however, say that all looked at it from that point of view. There was another class, the residuum, whose one idea in life was to get all they could for nothing, and to give as little in return as possible. That these were a difficulty was not to be denied, but they were not the only difficulty. It was nothing new in family life that the savings of all the respectable members should have to come to rescue one who was not respectable from consequences which were leading to his own ruin, and generally that one accepted what was done for him as being only his due. It was just the old charitable problem all over again: Were you first to help those who would not help themselves, or those who deserved it most? They were seldom the same people. The question, what they were to do for those who might have done something for themselves, and had not done it, was one which perplexed the Metropolitan Hospital Committee and many others, and they must face it as best they could. But they must always remember that those who appealed to them in that way were a minority, and that even if they could not be dealt with in a manner entirely satisfactory, it was not proved that the general system was wrong. Within a mile radius of the hospital there was population of 270,000 of the poorer classes. No other similar institution existed in the near neighbourhood. That was, to a certain extent, an advantage, because it gave their experiment a chance of being fairly tried; but, on the other hand, it threw upon the hospital such an immense burden as to increase the necessity for such appeals as they were now making to the liberality of the public.

